# Review of the stiletto fly genus *Actenomeros* Winterton & Irwin (Diptera, Therevidae, Agapophytinae)

**DOI:** 10.3897/zookeys.120.1615

**Published:** 2011-07-25

**Authors:** Shaun L. Winterton

**Affiliations:** California State Collection of Arthropods, California Department of Food & Agriculture, Sacramento, California, USA

**Keywords:** Asiloidea, Therevidae, Australia

## Abstract

The endemic Australian genus *Actenomeros* Winterton & Irwin, 1999b is reviewed. Three species are transferred from *Nanexila* Winterton & Irwin, 1999a: *Actenomeros aureilineata* (Winterton & Irwin, 1999a) **comb. n.**, *Actenomeros intermedia* (Winterton & Irwin, 1999a) **comb. n.** and *Actenomeros paradoxa* (Winterton & Irwin, 1999a) **comb. n.** A new species (*Actenomeros budawang* **sp. n.**) is described and figured from New South Wales. A key to species is presented.

## Introduction

The completely endemic Australasian stiletto fly (Diptera: Therevidae) fauna is composed of 370 described species in 26 genera, exclusively placed in two subfamilies, Agapophytinae and Therevinae (Winterton 2009, 2011). A key to genera of the region can be found in Winterton (2011). *Actenomeros* Winterton & Irwin, 1999b is a small genus of two described species endemic to eastern Australia, previously classified in the poorly defined *Taenogera* genus-group (Winterton et al. 1999b), but now classified in Agapophytinae (Winterton 2006, 2011). Placement of the genus based on morphological characters is problematic with analyses indicating either a close relationship with *Taenogerella* Winterton & Irwin (Winterton et al. 1999b) or a clade comprising *Taenogera* Kröber, 1912, *Johnmannia* Irwin & Lyneborg, 1989 and *Eupsilocephala* Kröber, 1912 (Lambkin et al. 2005). Morphological similarities between *Actenomeros* and *Nanexila* Winterton & Irwin, 1999a include the presence of subapical anteroventral setae on the hind femur, antennae with a short, cylindrical scape and conical flagellum, flattened frons, wing cell m3 open, and lack of velutum patches on the femora and gonocoxites. Winterton et al. (1999a) erected the genus *Nanexila* with three species groups. One of these species groups (i.e. *Nanexila atricostalis* species group) was considered very different from the other members of the genus, but the lack of males for most species precluded the erection of a separate genus. It was noted though in Winterton et al. (1999b) that females of this species group (specifically *Nanexila paradoxa* Winterton & Irwin, 1999a) were similar to *Actenomeros*. Males are now known for *Nanexila paradoxa* and have the key taxonomic features of *Actenomeros*. The generic concept of *Actenomeros* is revised slightly in light of this; synapomorphies for the genus include multiple rows of postocular macrosetae in both sexes, and greatly reduced or absent articulated gonocoxal processes in the male (Winterton et al. 1999b). The gonocoxite has a large horn-like process in the males of the two previously described species of *Actenomeros* but are absent in the new species described herein (*Actenomeros budwang* sp. n.) and three species transferred to *Actenomeros* from *Nanexila* [i.e. *Actenomeros aureilineata* (Winterton & Irwin, 1999a) comb. n., *Actenomeros intermedia* (Winterton & Irwin, 1999a) comb. n. and *Actenomeros paradoxa* (Winterton & Irwin, 1999a) comb. n.]; this character is no longer considered synapomorphic for the genus. The discovery of this new species described herein from New South Wales increases the number of species in *Actenomeros* to six.

## Material and methods

Adult morphological terminology follows McAlpine (1981) as modified by Winterton et al. (1999a) and Winterton (2006). Genitalia were macerated in 10% KOH at room temperature for one day to remove soft tissue, then rinsed in distilled water and dilute acetic acid, and dissected in 80% ethanol. Preparations were then placed into glycerine, with images made with the aid of a digital camera mounted on a stereomicroscope. Specimen images at different focal points were taken using a digital camera and subsequently combined into a serial montage image using Helicon Focus (©HeliconSoft). Genitalia preparations were placed in glycerine in a genitalia vial mounted on the pin beneath the specimen.

The following collection acronyms are cited in the text:

CASCalifornia Academy of Sciences, San Francisco, California, USA
            

ANICAustralian National Insect Collection (Canberra)
            

ASCUNew South Wales Dept of Agriculture, Orange Agricultural Institute, Agricultural Scientific Collections Unit
            

## Taxonomy

### Actenomeros

Winterton & Irwin, 1999b

http://species-id.net/wiki/Actenomeros

#### Type species:

 *Actenomeros corniculaticaudus* Winterton & Irwin, 1999b: 280.

#### Diagnosis.

Head sub-spherical; frons grey to gold pubescent; minute, dark setae sometimes present; frons flat to rounded, width sexually dimorphic, male frons narrower, but eyes not contiguous; occiput concave; two-three poorly defined rows of postocular macrosetae, rarely a single row in female; antenna length shorter than head; scape and pedicel short cylindrical, with strong dark setae; flagellum conical, compressed laterally, style terminal; sternopleuron glabrous medially; legs pale yellow, tarsi darkened distally; mid coxa without setae on posterior surface; hind femur with dark, anteroventral setae sub-apically; fore and hind femora without velutum patches; scutal chaetotaxy: np, 3–4; sa, 2; pa, 1; dc, 2–4; sc, 1; wing cell m3 open; abdomen black, male often with extensive abdominal velutum, female often with triangular patches of velutum laterally on segments; male genitalia without velutum patches on ventral surface of gonocoxites; gonocoxite with outer process present, often long, upward directed and horn-like; articulated gonocoxal process greatly reduced or absent; ventral lobe of gonocoxite sometimes greatly enlarged; hypandrium triangular, glabrous, fused to gonocoxites laterally; gonocoxal apodeme relatively short; distiphallus narrow, straight; dorsal apodeme of parameral sheath ‘T’-shaped; ejaculatory apodemes relatively small, narrow; ventral apodeme forked; female genitalia with A1 and A2 acanthophorite spines well developed; tergite 8 with narrow process on anterior margin; furca sclerotized in a narrow ring; three spherical spermathecae; spermathecal sac shape trilobate, spermathecal duct arrangement paired, one spermathecal duct joining to each spermathecal sac duct or rarely alternating along common spermathecal sac duct (*Actenomeros intermedia*).

#### Comments.

Winterton et al. (1999b) noted that this genus superficially resembles *Nanexila* but is differentiated by 2–3 rows of postocular setae and male genitalic features such as a reduced or absent articulated gonocoxal process and sometimes a large horn-shaped outer process on the gonocoxite. The remaining species in the *Nanexila atricostalis* species-group (i.e. *Nanexila atricostalis* Winterton & Irwin and *Nanexila jimrodmani* Winterton) retained in *Nanexila* have a well-formed articulated gonocoxal process and can be distinguished from *Actenomeros* species using the key to Australasian genera in Winterton (2011). The horn-like process on the gonocoxites is only present in *Actenomeros corniculaticaudus* and *Actenomeros onyx.* Males are unknown for *Actenomeros aureilineata* comb. n.and *Actenomeros intermedia* comb. n.; females of these two species are described by Winterton et al. (1999b).

#### Included species.

*Actenomeros aureilineata* (Winterton & Irwin) comb. n., *Actenomeros budawang* sp. n., *Actenomeros corniculaticaudus* Winterton & Irwin, *Actenomeros intermedia* (Winterton & Irwin) comb. n., *Actenomeros onyx* Winterton & Irwinand *Actenomeros paradoxa* (Winterton & Irwin) comb. n.

#### Key to *Actenomeros* species

**Table:** 

1	Wing with costal area dark infuscate; scutal pubescence brown with gold medial stripe	*Actenomeros aureilineata* (Winterton & Irwin) comb. n.
–	Wing hyaline or at most, uniformly smoky infuscate	2
2	Postocular macrosetae with anterior row black, posterior row yellowish; single pair of supra-alar macrosetae; three notopleural macrosetae	*Actenomeros intermedia* (Winterton & Irwin) comb. n.
–	Postocular macrosetae black; two pairs of supra-alar macrosetae; four or more notopleural macrosetae	3
3	Gonocoxite without enlarged, ‘horn’-like process; articulated gonocoxal process absent	4
–	Gonocoxite with greatly enlarged, ‘horn’-like process posterolaterally; articulated gonocoxal process present, greatly reduced	5
4	Setae along costal margin short, length approximately equal to width of costal vein; posterior margin of scutellum with yellow margin (sometimes faint); male terminalia yellow with brown medially; ventral lobe of gonocoxite elongate, rounded (Fig. 5 F-G)	*Actenomeros paradoxa* (Winterton & Irwin) comb. n.
–	Setae along costal margin elongate, length approximately twice width of costal vein; scutellum uniform grey pubescent; male terminalia brown-black; ventral lobe of gonocoxite shorter, anvil shaped (Fig. 5 B-C)	*Actenomeros budawang* sp. n.
5	Male gonostylus with two narrow, ventrally directed processes, one basal and the other distal	*Actenomeros corniculaticaudus* Winterton & Irwin
–	Male gonostylus with single, ventrally directed process near apex	*Actenomeros onyx* Winterton & Irwin

### Actenomeros budawang sp. n.

urn:lsid:zoobank.org:act:E80EF2AB-394D-44C6-B70C-32DAAF665FE5

http://species-id.net/wiki/Actenomeros_budawang

**Figure 1.**
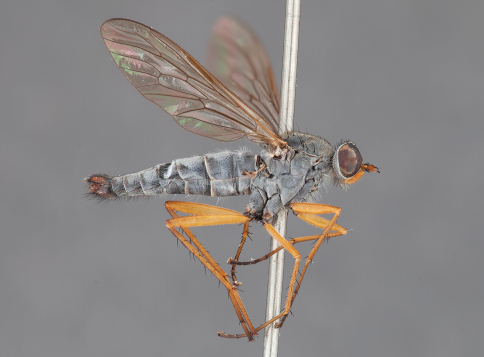
*Actenomeros budawang* sp. n. Male habitus, lateral. Body length = 8.5 mm.

Figs 1 [Fig F2] [Fig F3] –4 , 5A-E, H 

#### Type material.

 **Holotype** male, AUSTRALIA: **New South Wales**: Budawang National Park, ca. 5km on Western Distributor Road, 250m asl, MV lamp & UV fit, [-35.334, 150.034], 22.ix.2004, A. Zwick (ANIC).

**Paratypes**. AUSTRALIA: **New South Wales**: female, same data as holotype (CAS); male, 2 km W Thirlmere Lakes National Park, 25.ix.1988, G.R. Brown, M.A.Terras [-34.228, 150.536] (ASCU); 4 males, Warrumbungle National Park, Wambelong Creek, [-31.323, 149.027], 21.i–9.ii.2009, Malaise trap across creek, S.L. Winterton (CAS).

#### Diagnosis.

Setae along costal margin elongate, length approximately twice width of costal vein; scutum uniform grey pubescent; articulated gonocoxal process completely absent; process on gonocoxite straight, elongate, not horn-like; ventral lobe of gonocoxite relatively short, anvil shaped, female with two rows of dark postocular macrosetae.

#### Description.

Body length: 8.0–9.5 mm [male]; 10.0 mm [female]. *Head*: Frons gold pubescent, short dark setae present in female, male frons narrower than anterior ocellus and narrowest point; occiput grey pubescent, postocular ridge with 2–3 poorly defined rows of black setae in both sexes; gena grey pubescent, admixed with fine dark setae; parafacial grey pubescent, without setae; mouthparts pale orange; scape and pedicel yellow, combined length approximately equal to flagellum length, numerous strong, dark setae present except on medial surface (Fig. 5H); flagellum with 3 segments, yellow with dark suffusion dorsally and distally, without dark setae on basal flagellomere; style dark. *Thorax*: Scutum and scutellum grey pubescent with three irregular brown pubescent stripes, numerous fine dark setae scattered over surface, longer in male; pleuron and coxae grey pubescent; fine pale setae sparsely scattered over proepisternum, anepisternum, katepisternum, pteropleural callus and coxae; strong dark macrosetae on anterior surface of coxa; legs pale yellow, trochanters brown, tarsi darkened distally; halter dark yellow to brown; wing uniformly smoky infuscate, venation dark; setae along costal margin elongate, length approximately twice width of costal vein. Scutal chaetotaxy: np, 4–5; sa, 2 (rarely 3); pa, 1; dc, 3–5; sc, 1 (rarely 2). *Abdomen*: Abdomen glossy brown-black, male with extensive silver velutum on segments 1–7, reduced to posterior margins of tergites 2–6 in female; numerous fine, white setae on all segments, shorter in female; terminalia brown with black setae. *Male Genitalia* (Fig 5A-E): Epandrium elongate, sub-quadrangular, narrowed posteriorly, numerous strong, dark setae laterally; posterior margin of tergite 8 medially emarginate, posteriorly directed setae on posterolateral corners; hypandrium triangular, relatively small, fused to gonocoxites laterally; gonocoxite with strong dark setae over outer surface; ventral lobe dark sclerotized with ventral directed process apically; gonocoxal apodeme relatively short; posteriorly directly process of gonocoxite straight, narrowed apically; articulated gonocoxal process completely absent; gonostylus well developed with strong spinose processes laterally and dorsally, setae along lateral surface; distiphallus straight, ridged like dorsally; dorsal apodeme broadly T-shaped; minute spines on distiphallus and dorsal apodeme; lateral ejaculatory apodeme relatively small, narrow. Female genitalia: tergite 8 with narrow process on anterior margin; furca sclerotized in a narrow ring, spermathecal sac shape trilobate as in figure 5I, spermathecal duct arrangement paired, one spermathecal duct joining to each spermathecal sac duct.

#### Etymology.

This species is named after the type locality, Budawang National Park, in central-southern New South Wales.

#### Comments.

*Actenomeros budawang* sp. n. is similar in appearance to *Actenomeros paradoxa* comb. n., suggesting a likely close relationship. The former can be distinguished by the greatly enlarged ventral lobe, lack of ‘horn’-like gonocoxal process and complete absence of the articulated gonocoxal process. The scutal chaetotaxy is variable in this species *Actenomeros budawang* sp. n.

**Figure 2. F2:**
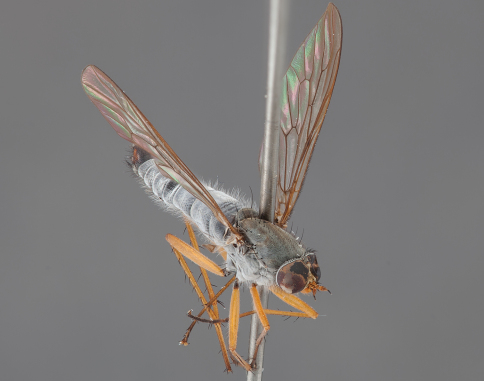
*Actenomeros budawang* sp. n. Male habitus, oblique view. Body length = 8.5 mm.

**Figure 3. F3:**
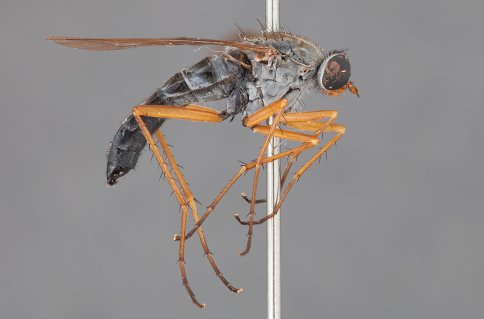
*Actenomeros budawang* sp. n. Female habitus, lateral. Body length = 10.0 mm.

**Figure 4.**
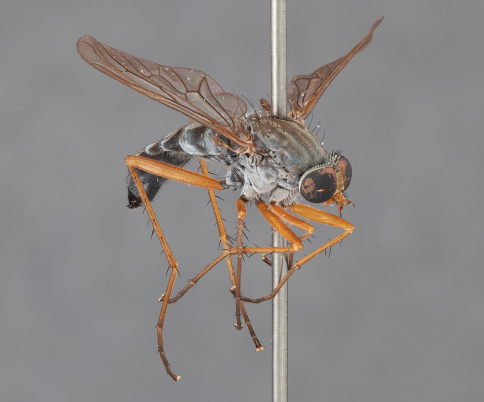
*Actenomeros budawang* sp. n. Female habitus, oblique view. Body length = 10.0 mm.

**Figure 5.**
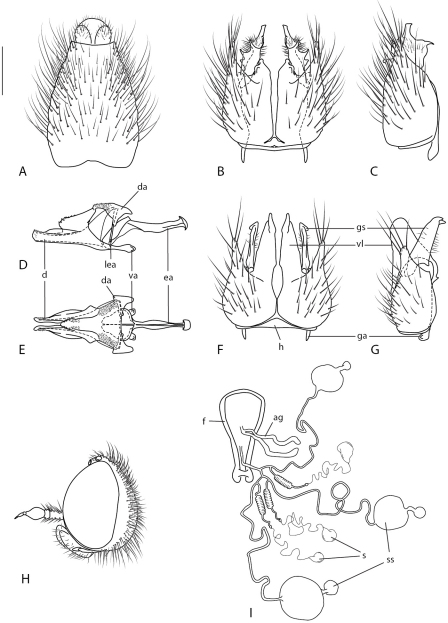
*Actenomeros* spp. *Actenomeros budawang* sp. n.: male genitalia: **A** epandrium, dorsal view **B** gonocoxites, ventral view **C** gonocoxite, lateral view **D** aedeagus, lateral view **E** aedeagus dorsal view. *Actenomeros paradoxa* (Winterton & Irwin) comb. n.: male genitalia: **F** gonocoxites, ventral view **G** gonocoxite, lateral view **H** *Actenomeros budawang* sp. n.: male head, lateral view. *Actenomeros paradoxa* (Winterton & Irwin) comb. n.: female internal genitalia, dorsal view. Abbreviations: *ag*, accessory gland; *c*, cercus; *d*, distiphallus; *da*, dorsal apodeme of parameral sheath; *ea*, ejaculatory apodeme; *f*, furca; *ga*, gonocoxal apodeme; *gs*, gonostylus; *h*, hypandrium; *lea*, lateral ejaculatory apodeme; *s*, spermatheca; *ss*, spermathecal sac; *va*, ventral apodeme of parameral sheath; *vl*, ventral lobe. Scale line = 0.2 mm.

## Supplementary Material

XML Treatment for Actenomeros

XML Treatment for Actenomeros budawang
